# Extensive aggregation of α-synuclein and tau in juvenile-onset neuroaxonal dystrophy: an autopsied individual with a novel mutation in the *PLA2G6* gene-splicing site

**DOI:** 10.1186/2051-5960-1-12

**Published:** 2013-05-09

**Authors:** Yuichi Riku, Takeshi Ikeuchi, Hiroyo Yoshino, Maya Mimuro, Kazuo Mano, Yoji Goto, Nobutaka Hattori, Gen Sobue, Mari Yoshida

**Affiliations:** 1 Department of Neurology, Nagoya Daiichi Red Cross Hospital, Aichi, Japan; 2 Department of Neurology, Nagoya University Graduate School of Medicine, Aichi, Japan; 3 Department of Neurology, Brain Research Institute, Niigata University, Niigata, Japan; 4 Research Institute for Diseases of Old Age, Graduate School of Medicine, Juntendo University, Tokyo, Japan; 5 Institute for Medical Science of Aging, Aichi Medical University, Aichi, Japan; 6 Department of Neurology, Graduate School of Medicine, Juntendo University, Tokyo, Japan

**Keywords:** α-synuclein, Infantile neuroaxonal dystrophy, Atypical neuroaxonal dystrophy, PLA2G6 gene, Tau

## Abstract

**Background:**

Infantile neuroaxonal dystrophy (INAD) is a rare autosomal-recessive neurodegenerative disorder. Patients with INAD usually show neurological symptoms with infant onset and die in childhood. Recently, it was reported that mutations in the *PLA2G6* gene cause INAD, but neuropathological analysis of genetically confirmed individuals with neuroaxonal dystrophy has been limited.

**Results:**

Here, we report a Japanese individual with neuroaxonal dystrophy associated with compound heterozygous mutations in the *PLA2G6* gene. A novel splice-site mutation resulting in skipping and missense mutations (p.R538C) in exon 9 was identified in the patient. This patient initially presented with cerebellar ataxia at the age of 3 years, which was followed by symptoms of mental retardation, extrapyramidal signs, and epileptic seizure. The patient survived until 20 years of age. Neuropathological findings were characterized by numerous axonal spheroids, brain iron deposition, cerebellar neuronal loss, phosphorylated alpha-synuclein-positive Lewy bodies (LBs), and phosphorylated-tau-positive neurofibrillary tangles. In particular, LB pathology exhibited a unique distribution with extremely severe cortical involvement.

**Conclusions:**

Our results support a genetic clinical view that compound heterozygous mutations with potential residual protein function are associated with a relatively mild phenotype. Moreover, the severe LB pathology suggests that dysfunction of the *PLA2G6* gene primarily contributes to LB formation.

## Background

Neurodegeneration with brain iron accumulation (NBIA) describes a group of progressive neurodegenerative disorders that are pathologically characterized by the presence of axonal spheroids and iron deposition in the brain [1-3]. These neurodegenerative diseases consist of a clinically and genetically heterogeneous group of disorders, including pantothenate kinase-associated neurodegeneration (PKAN, formerly known as Hallervorden-Spatz disease), infantile neuroaxonal dystrophy (INAD), and an unknown gene mutation-linked idiopathic neuroaxonal dystrophy [1,2,4]. PKAN is caused by mutations in the pantothenate kinase 2 (*PANK2*) gene, which accounts for the majority of NBIA patients [2]. Recently, it was reported that mutations in the phospholipase A2 group VI (*PLA2G6*) gene cause INAD [5], which is a rare autosomal-recessive neurodegenerative disorder. Patients with INAD usually present with psychomotor regression, truncal hypotonia, progressive ataxia, extrapyramidal symptoms, fast waves on an electroencephalogram, and neuro-ophthalmological abnormalities (e.g., optic atrophy, nystagmus, and strabismus) with infant onset and die in childhood [1,4,6]. However, in rare cases, patients with NAD caused by *PLA2G6* mutations present with heterogeneous neurological manifestations with onset past infanthood and survive until adulthood with a slower disease progression [1,7,8]. In addition, mutations of the *PLA2G6* gene cause early onset dystonia-parkinsonism (PARK-14), which is clinically distinguished from NAD by good L-dopa responsiveness, L-dopa–induced dyskinesia, and dementia. These characteristics have been typically observed in patients with an older age of onset and with a longer disease duration compared to NAD, with no evidence of cerebellar symptoms [9]. Thus, these clinical phenotypes are collectively termed as *PLA2G6*-associated neurodegeneration [9].

We report a Japanese individual with neuroaxonal dystrophy that was associated with a novel compound heterozygous mutation in a splicing site of the *PLA2G6* gene. The clinical phenotype of this patient was atypical for INAD, occurred during late disease onset, and prolonged the disease course. Histopathological data revealed the presence of neuroaxonal spheroids, brain iron depositions, and cerebellar degeneration. Moreover, numerous Lewy bodies (LBs) and neurofibrillary tangles (NFTs), which are pathological hallmarks of Parkinson’s disease (PD) and Alzheimer’s disease (AD), respectively, were observed. Until recently, neuropathological analysis of genetically confirmed neuroaxonal dystrophy has been strongly limited due to a small number of patients [1,8]. In this study, we describe the clinicopathological characteristics of the patient and discuss the neuropathological implication of LBs and NFTs compared with PD and AD.

## Case presentation

### Clinical history

**Figure 1:**
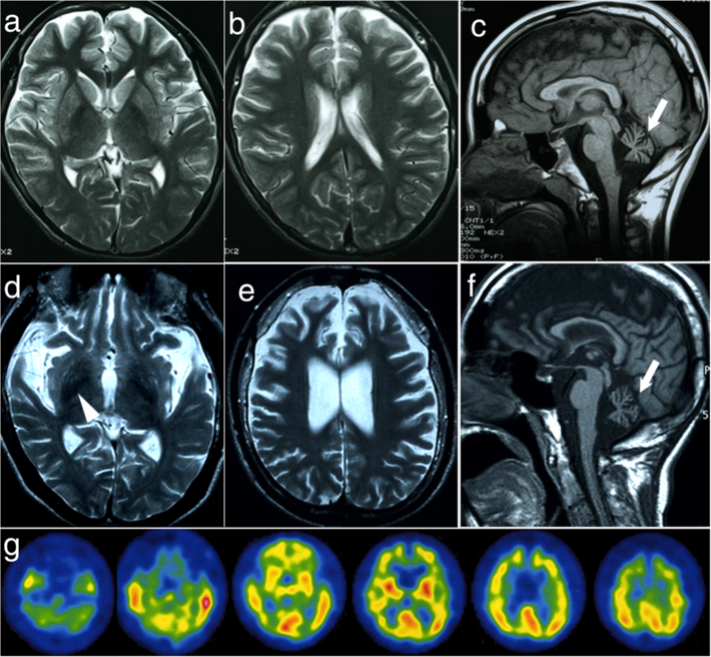
**Magnetic resonance imaging (MRI) and Tc99m-ECD-single-photon emission computed tomography (SPECT) of the patient. a-c** MRI at age 12. There was mild atrophy of the frontal cortex and slightly low intensity in the globus pallidus on T2-weighted images (T2WI) (**a, b**). The saggital section of the T1WI exhibited cerebellar atrophy (arrow) (**c**). **d-f** MRI at age 18. Low signal intensity in the globus pallidus (arrowhead) and atrophy of the temporal lobes was clear on the T2WI (**d**). The frontal lobes showed severe atrophy (**e**). The saggital section of the T1WI exhibited severe cerebellar atrophy (arrow) and thinness of the corpus callosum (**f**). An ECD-SPECT, at age 18, revealed hypoperfusion of the frontotemporal lobes and cerebellum (**g**).

The patient was a Japanese man who died at 20 years of age. He exhibited normal development until the age of 3 years, at which time his parents noted his slurred speech and unstable gait. There was no evidence of a consanguineous marriage in any of his relatives. His grand-aunt had been diagnosed with “parkinsonism”, and she died at the age of 60; however, her clinical diagnosis was uncertain. At the age of six, the patient was referred to our hospital due to a progressive gait disturbance and dysarthria. A neurological examination revealed cerebellar ataxia, bradykinesia, mental retardation, and hyperreflexia in the lower limbs without pathological reflexes. Truncal hypotonia and abnormalities in eye movement were not observed. Cerebral computed tomography (CT) showed severe cerebellar atrophy. The patient was clinically diagnosed with juvenile spinocerebellar degeneration, and taltirelin was administered for his ataxia; however, it did not have an effect. At the age of 12, cerebral magnetic resonance imaging (MRI) revealed severe atrophy of the cerebellum and mild atrophy of the frontal lobes (Figure 1a-c). The patient gradually became bedridden until the age of 15 and started experiencing repetitive generalized seizures. He was mainly treated with sodium valproate and phenobarbital. At the age of 18, he was re-admitted to our hospital, although he was nearly bedridden and could barely sit in a wheelchair at that time. Neurological examination revealed severe dystonia and rigidity in his limbs and neck, a masked face, and severe cerebellar ataxia. His tendon reflexes showed hyperreflexia in the upper limbs and were abolished in his lower limbs. Moreover, his plantar responses were flexor. CT and MRI (Figure 1d-f) revealed severe cerebellar and fronto-temporal lobe atrophy. The cerebral atrophy was more progressive compared to the atrophy observed when he was 12 years old. By T2-weighted imaging (T2WI), the bilateral globus pallidus (GP) and putamen exhibited low signal intensity. Tc99m-ECD-single-photon emission computed tomography revealed hypoperfusion in the fronto-temporal lobes and cerebellum (Figure 1g). An electroencephalogram showed multifocal spikes and theta waves in the right hemisphere in the absence of fast waves. The results of the nerve conduction study on the four limbs were normal. After discharge, a higher dose of valproate reduced the frequency of the patient’s seizures; however, his rigidity and dystonia showed no response to L-DOPA treatment. The patient died of aspiration pneumonia.

### Materials and methods

#### Neuropathological analysis

The postmortem interval was 5 hours. The brain and spinal cord were fixed in 20% neutral formalin. Samples obtained from the main representative regions of the brain and spinal cord were embedded in paraffin, sectioned into 4.5-μm-thick slides, and stained with hematoxylin and eosin (H&E), Klüver-Barrera staining, Prussian blue methods, and Gallyas-Braak (GB) staining. Immunohistochemical studies were performed on 4.5-μm-thick sections using an ENVISION kit (Dako) with diaminobenzidine (DAB; Wako, Osaka, Japan) as a chromogen. The primary antibodies used were anti-phosphorylated alpha-synuclein (p-α-synuclein) (pSyn#64, monoclonal mouse, 1:1000; Wako Pure Chemical Industries, Osaka, Japan), anti-ubiquitin (polyclonal rabbit, 1:2000; Dako), anti-amyloid-beta peptide (6F/3D, monoclonal mouse, 1:200; Dako), phosphorylated tau (p-tau) (AT8, monoclonal mouse, 1:2000; Innogenetics, Zwijndrecht, Belgium), anti- TDP-43 (TARDBP, polyclonal rabbit, 1:2500; ProteinTech, IL, USA), and anti-phosphorylated neurofilament (p-NF) (2F11, monoclonal mouse, 1:600; Dako). For double-immunofluorescence labeling, brain tissues obtained from the amygdala, oculomotor nucleus, and substantia nigra were sectioned into 4.5-μm-thick slides. The primary antibodies were anti-p-α-synuclein antibody and AT8 antibody. The secondary antibodies were goat anti-mouse IgG coupled with either Alexa Fluor 568 (1:300, emission peak 603 nm, Molecular Probes, OR, USA) or Alexa Fluor 488 (1:300, emission peak 517 nm, Molecular Probes). The slides were examined via confocal microscopy at × 200 and × 400 magnification using a Zeiss LSM 710 laser scanning confocal microscope.

For electron microscopy, sections from the cingulate gyrus were fixed in 4% glutaraldehyde. The sections were washed in phosphate buffer, postfixed with osmium tetroxide, dehydrated in a graded series of ethanol, and embedded in Epon. Ultrathin sections were stained with uranyl acetate and lead citrate.

#### Western blotting analysis of α-synuclein

Proteins expressed in the amygdala and parahippocampal gyrus of the autopsied patient and three control subjects were extracted as previously described [10,11]. Briefly, we fractionated the samples by resolubilization in increasingly stringent buffers (Tris-buffered saline, 1% Triton X-100, 1% sarcosyl, 8 M urea) as previously described. Equal amounts of supernatant protein were subjected to sodium dodecyl sulfate-polyacrylamide gel electrophoresis and immunoblotting. The mouse monoclonal antibody LB509 (Zymed Laboratories, South San Francisco, California) was used to detect α-synuclein. The monoclonal antibody pSyn#64 (Wako, Japan) specifically recognizes phosphorylated α-synuclein at serine 129 [12].

#### Genetic analysis

Genomic DNA was extracted from the frozen liver tissue of the patient using a standard procedure. Mutational analysis was performed using sequences of both strands of all of the PCR-amplified coding exons and the flanking intronic sequences of *PLA2G6, PANK-2, SNCA*, *parkin*, *PINK-1*, and *DJ-1.* Expansion of the CAG repeats of the SCA1, SCA3, DRPLA, and Huntington’s disease genes was also examined. Genetic analysis of *PLA2G6* was also performed in the patient’s parents. Total RNA was isolated from frozen brain tissue of the patient, and cDNA was synthesized using a High-Capacity cDNA Reverse Transcription kit (Applied Biosystems). RT-PCR was performed using primer pairs to amplify the coding regions of the *PLA2G6* gene spanning exons 8–13 (5′-caacgtggagatgatcaagg-3′ and 5-gtcagcatcaccttgggttt-3′) and exons 9–13 (5′-ggaaggcgatcttgactctg-3′ and 5′-gtcagcatcaccttgggttt-3′). The institutional review board approved this study.

### Results

#### Neuropathological findings

**Figure 2:**
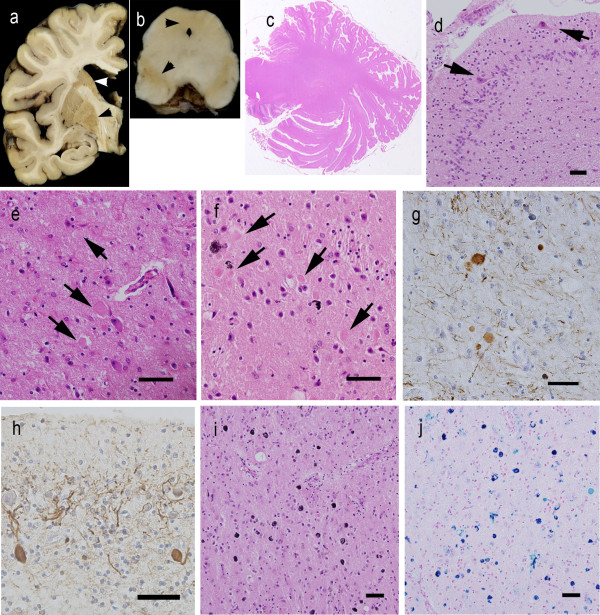
**The macroscopic and microscopic findings in the patient. a, b** The globus pallidus, putamen, caudate, substantia nigra, and periaqueductal gray matter demonstrated yellow-brown discoloration (arrowheads). **c** Grossly, the cerebellar cortex showed severe atrophy, and the granule cell layer was not visible. **d** The cerebellar granule cells were markedly depleted, and ectopic Purkinje cells (Pcs) were found (arrows). **e** The cingulate gyrus showed gliosis and numerous axonal spheroids (arrows). **f, g** The putamen also contained numerous axonal spheroids that were labeled by anti-phosphorylated neurofilament antibody. **h** In the cerebellum, anti-phosphorylated neurofilament immunostaining revealed dystrophic axons of the Pcs and highly reduced parallel fibers. **i** Many iron-positive granules were observed in the putaminal neuropil. **j** These granules were evident after Prussian blue staining. Bar = 50 μm. Hematoxylin and eosin staining (**c-f, i**), phosphorylated neurofilament immunohistochemistry (**g, h**), and Prussian-blue staining (**j**).

The patient’s height was 150 cm, and his body weight was 39 kg. The brain weighed 890 g before fixation. Grossly, the cerebral hemispheres showed severe atrophy, particularly in the fronto-temporal cortex. In the brain sections, the most striking pathological finding was a light yellow-brown discoloration of the substantia nigra (SN), periaqueductal gray matter, putamen, caudate head, and GP (Figure 2a,b). The cerebellum was greatly reduced in size; its overall convolution pattern was retained, but the individual folia were shrunken.

Histopathologically, severe neuronal loss and gliosis were observed in the cerebral cortex, brainstem gray matter, and cerebellar cortex. In the cerebrum, neuronal loss was marked in the cingulate gyrus, fronto-temporal cortex, insular cortex, amygdala, and hippocampus. In the brain stem, neurons in the SN were markedly depleted, and the remaining neurons had low melanin content. The locus ceruleus (LC) showed moderate neuronal loss, but the neurons in the dorsal motor nucleus of the vagus were relatively spared. The cerebellar cortex showed severe neuronal loss (Figure 2c,d), particularly in the granule cell (gc) layer, and the parallel fibers in the molecular layer were strongly reduced (Figure 2c,d,h). Purkinje cells (Pcs) were severely depleted and often ectopically scattered at random in the molecular layer (Figure 2d). The spinal cord exhibited myelin pallor of the gracile fasciculus with gliosis.

In addition, we observed axonal spheroids throughout the central nervous system (CNS), particularly in the cerebral cortex, putamen (Figure 2e-g), caudate nucleus, nucleus accumbens, hypothalamus, SN, gracile nucleus, and spinal cord. The cerebellum contained numerous dystrophic axons called ‘torpedoes’ in the Pc and gc layers (Figure 2h). The diameters of the spheroids ranged from 10 to 20 μm, but the spinal cord contained larger-sized spheroids of 40–70 μm in diameter. Various spheroids were immunoreactive against anti-p-NF (Figure 2g,h) and anti-ubiquitin antibodies. We found no spheroids in the sympathetic ganglia, dorsal root ganglia, spinal roots, peripheral nerve fibers in the skin, or the enteric plexus. Brown-pigmented, Prussian blue-positive iron granules were scattered around the vessels and throughout the neuropil in the putamen, internal segment of the GP, caudate nucleus, thalamus, pars compacta of the SN, and periaqueductal gray matter (Figures 2i,j, 4c).

**Figure 3:**
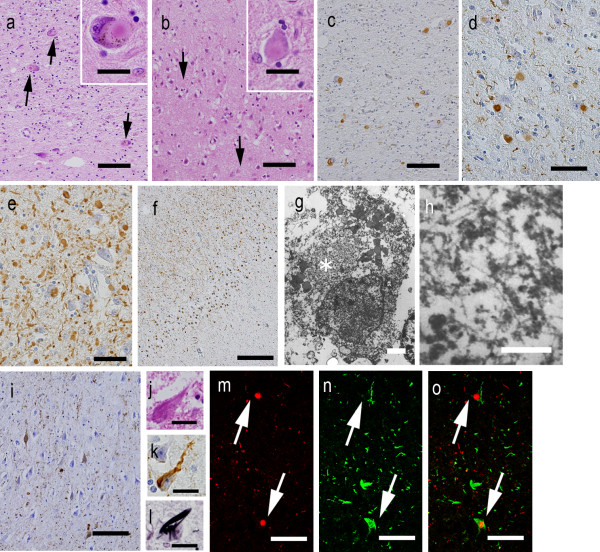
**Lewy body (LB) and neurofibrillary tangle (NFT) pathology of the patient. a and inset** The substantia nigra contained “brainstem-type” LBs with core and halo (arrows) structures. **b and inset** In the cingulate gyrus, the neuropil showed spongy changes in the deep layer, and there were “cortical-type” LBs without core and halo structures (arrows). **c and d** Anti-phosphorylated alpha-synuclein (p-α-synuclein) immunohistochemistry showed abundant LBs in the substantia nigra (**c**) and cingulate gyrus (**d**). **e and f** In the hypothalamus (**e**) and Ammon’ s horn (**f**), p-α-synuclein-positive LBs and LNs were strikingly abundant. **g and h** Electron microscopy of a neuron in the cingulate gyrus showed cortical LBs in a cortical neuron (asterisk), which consisted of granular and filamentous structures. The filaments were arranged at random without a clear central zone density. **i - l** The pyramidal neuron in the hippocampal cortex contained abundant NFTs and threads that were positive for AT-8 antibody using the Gallyas-Braak method. **m-o** Confocal microscopy of the amygdala revealed immunoreactivity against p-α-synuclein (**m**, red), which often co-labeled with AT8 (**n**, green) in the same neurons (**o**, merged). Bar (**a**), (**b**), (**c**), and (**i**) = 100 μm; (**d**), (**e**), (**m**), (**n**), and (**o**) = 50 μm; (**f**) = 250 μm; (**g**) = 2 μm; (**h**) = 0.2 μm; (**a-inset**), (**b-inset**), (**j**), (**k**), and (**l**) = 20 μm. Hematoxylin and eosin staining (**a, b, j**), p-α-synuclein immunohistochemistry (**c-f**), AT8 immunohistochemistry (**i, k**), and Gallyas-Braak staining (**l**).

**Figure 4:**
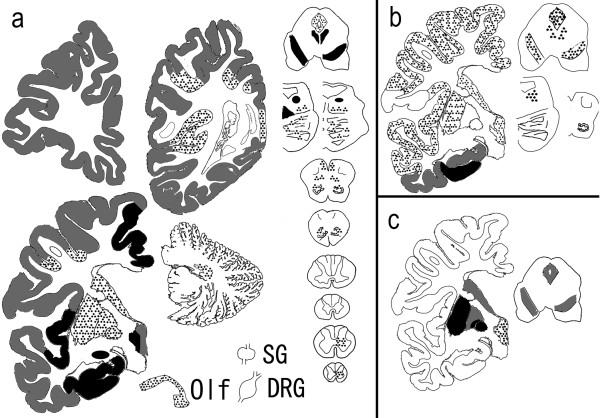
**Schema of the distribution of Lewy bodies (LBs), neurofibrillary tangles (NFTs), and iron deposition in the patient. a** LBs were diffusely spread in the cerebral cortices and were most abundant in the limbic system, hypothalamus, nucleus basalis of Meynert, substantia nigra (SN), and locus ceruleus. The olfactory bulbs and dorsal motor nucleus of the vagus contained only mild LB pathology. There were no p-α-synuclein-positive structures in the peripheral sympathetic ganglia and dorsal root ganglia. **b** NFTs were predominantly observed in the limbic system, which corresponded to Alzheimer’s disease in Braak stage IV. **c** Iron-positive granules were found in the putamen, internal segment of the globus pallidus, caudate nucleus, SN, and periaqueductal gray matter. *DRG*: dorsal root ganglia, *Olf*: olfactory bulb, *SG*: sympathetic ganglia. Semi-quantitative scale for LBs and NFTs: *dot-pattern* = 1–10, *gray* = 10–20, *black* > 20. Number of lesions in a field observed using a 10× objective. Iron-positive granules: *dot-pattern* = mild, *gray* = moderate, *black* = severe.

Furthermore, severe LB pathology was observed throughout the brain (Figures 3a-f, 4a). In immunohistochemistry, anti-p-α-synuclein (Figure 3c-f), anti-p-NF, and anti-ubiquitin antibodies strongly labeled LBs. Immunohistochemistry using anti-p-α-synuclein antibody also revealed numerous dilated and sausage-like dystrophic neurites in the neuropil, which have been referred to as Lewy neurites (LNs) (Figure 3d,e). In the cerebral cortices, numerous cortical-type LBs, which lacked core and halo structures, and LNs were diffusely found (Figure 3b,e) with striking spongiform alterations (Figure 3b). The distribution and density of LBs and LNs exceeded what has been observed in advanced PD or DLB in the neocortical stage [13,14]. They were most abundant in the cingulate gyrus, amygdala, anterior hippocampus, CA2 region of the posterior hippocampus, and hypothalamus. LBs with a core and halo (brainstem-type LBs) were observed predominantly in the nucleus basalis of Meynert, SN, oculomotor nucleus, and LC (Figure 3a,c). In the cerebellum, neuronal cytoplasm and neurites of the dentate nucleus rarely contained p-α-synuclein-positive structures. In contrast, the olfactory bulbs and dorsal motor nucleus of the vagus contained only mild LB pathology. There were no p-α-synuclein-positive structures in the peripheral sympathetic ganglia, dorsal root ganglia, cardiac sympathetic nerve fibers, or nerve plexuses in the gastroenteric organs. Numerous p-tau-positive NFTs were also found predominantly in the limbic system (Figures 3i-l, 4b). The abundance of NFTs corresponded to AD in Braak stage IV [15], however, Aβ-positive neuritic plaques and amyloid deposits were absent. Additionally, TDP-43-positive inclusions were absent in this patient.

Using double-immunofluorescence labeling in the amygdala, p-tau-positive NFTs and p-α-synuclein-positive cortical LBs often co-labeled in the same neuron (Figure 3m-o). In contrast, neurons in the midbrain did not show co-labeling of LBs and NFTs (data not shown).

On electron microscopy, LBs from the cingulate gyrus consisted of closely packed 6- to 10-nm-thick granular and filamentous structures. The filaments were arranged at random without a clear central zone density. These findings were similar those observed in cortical-type LBs in the DLB [16] (Figure 3g,h).

#### Western blotting analysis of α-synuclein

**Figure 5:**
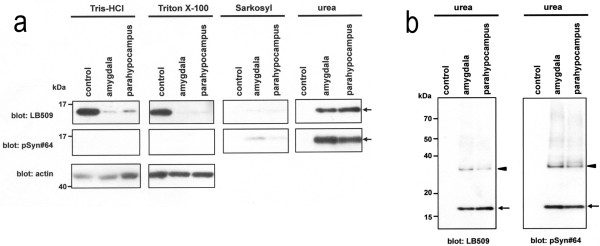
**Biochemical analysis of α-synuclein in brain tissues. a** The samples were sequentially fractionated by resolubilization with increasingly stringent buffers containing Tris-buffered saline, Triton X-100, sarkosyl, and urea. The samples were analyzed using the α-synuclein antibodies LB509 and pSyn#64. In the urea-extracted fraction, a phosphorylated α-synuclein band at approximately at ~15 kDa (arrow) was substantially accumulated in the brain (amygdala and parahippocampus) of the autopsied patient. Conversely, the level of α-synuclein in the Tris- and Triton X-100-soluble fractions was decreased. Anti-β-actin immunoblotting served as a loading control (bottom panel). **b** α-Synuclein-reactive fragments of dimers migrating at approximately ~30 kDa (arrowhead) and a smear at higher molecular weights were abundant in the urea-extracted fraction of the patient’s brain.

To biochemically characterize the accumulation of α-synuclein, we sequentially extracted proteins from the amygdala and parahippocampal gyrus of the autopsied male subject using buffers with increasing capacities to solubilize proteins. In the control sample, immunoblotting analysis using anti-α-synuclein LB509 showed an approximately 15-kDa band corresponding to monomeric α-synuclein in Tris–HCl- and Triton X-100-soluble fractions (Figure 5a). In contrast, an ~15-kDa α-synuclein band was predominantly visualized in sarkosyl-insoluble urea-soluble fractions in the brain (Figure 5a). In addition to the ~15-kDa band, an ~30-kDa band corresponding to α-synuclein dimers was observed in the insoluble fractions extracted from the patient’s brain samples. Importantly, the ~15- and ~30-kDa bands found in the urea-extracted fractions were reactive to anti-pSyn#64 antibody (Figure 5b), indicating that the accumulated α-synuclein in the patient brain was phosphorylated at serine 129. Moreover, the patient showed large amounts of α-synuclein-reactive high-molecular-weight smears, which might have represented α-synuclein oligomers (Figure 5b).

#### Genetic analysis

**Figure 6:**
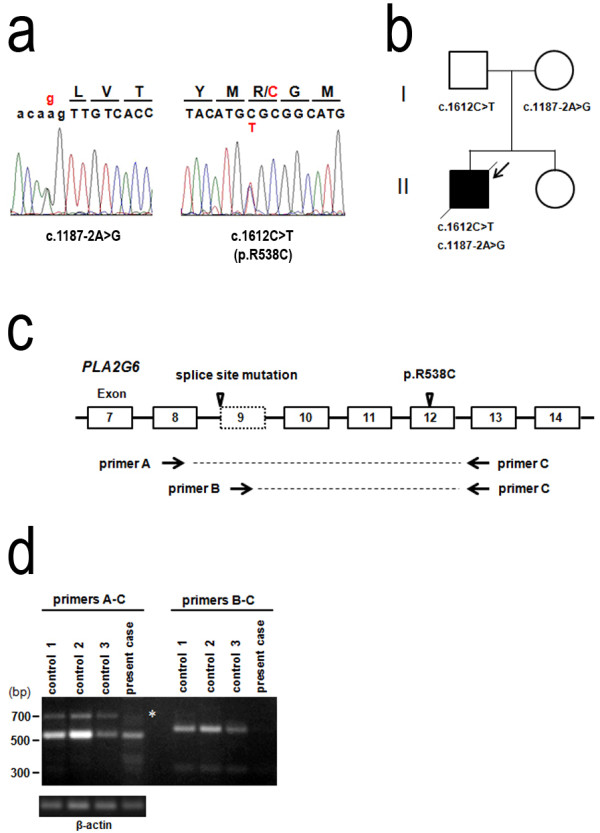
**Identification of compound heterozygous mutations in *PLA2G6 *in the patient. a** Mutation analysis of *PLA2G6* in the patient. Sequence analysis of the patient’s genomic DNA revealed two mutations: a 5′ splice-site mutation in exon 9 (c.1187-2A > G) and a missense mutation (c.1612C > T, p.R538C) in exon 12. The capital and small letters represent nucleotides in exons and introns, respectively. **b** Pedigree of the patient with *PLA2G6* mutations. Circle, female; square, male; slash through symbol, deceased individual; closed symbol, affected individual. An arrow denotes the proband. The father and mother each carried one of the compound heterozygous mutations found in the patient. **c** Schematic illustration of exon-intron structure of *PLA2G6*. Boxes represent exons. The positions of the mutations in the patient are shown. Two primer pairs were designed to amplify cDNA fragments encompassing exons 8 to 13 (primers A-C) and 9 to 13 (primers B-C). **d** Reverse transcription (RT)-PCR analysis of the patient’s brain. In the patient, a 700-bp fragment containing exon 9 was nearly undetectable by RT-PCR using the primer pair A-C. RT-PCR amplification using the primer pair B-C revealed a 600-bp cDNA fragment containing exon 9, which was recognized in control samples but was less noticeable in the autopsied patient. Amplification of β-actin mRNA was used as an internal control.

Sequence analysis revealed that the patient carried compound heterozygous mutations in the *PLA2G6* gene. A novel splice-site mutation in exon 9 (c.1187-2A > G) and a missense mutation (c.1612C > T, p.R538C) in exon 12 (Figure 6a) were identified in the patient. These mutations were absent in 120 normal control subjects. The p.R538C missense mutation was previously reported in patients with classical INAD [5]. Genetic analysis of the unaffected parents of the patient revealed that the father and mother were heterozygous carriers of the p.R538C missense and splice-site mutations, respectively (Figure 6b). No mutations were identified in *PANK2*, *SNCA*, *parkin*, *DJ-1*, *or PINK-1*. There was no pathological expansion of CAG repeats in the genes associated with SCA1, SCA2, DRPLA, or Huntington’s disease.

We next investigated whether the splice-site mutation caused an alteration in the splicing of *PLA2G6* using RT-PCR analysis of total RNA extracted from the patient’s frozen tissue. To examine the mRNA expression of *PLA2G6* in the patient’s brain, two primer pairs were designed to amplify cDNA fragments encompassing exons 8 to 13 (primers A-C) and 9 to 13 (primers B-C), respectively (Figure 6c). In the control samples, two alternative splicing variants were amplified using the primer pairs A-C. Sequence analysis of the fragments in the control samples revealed that these variants corresponded to two previously reported isoforms of *PLA2G6* mRNA, with and without the 162-bp exon 9, respectively [17]. In the patient, the 700-bp fragment containing exon 9 was nearly undetectable by RT-PCR using the primer pair A-C. RT-PCR amplification using the primer pair B-C revealed that the 600-bp cDNA fragment containing exon 9 found in the control samples was less expressed in the patient (Figure 6d).

### Discussion

INAD is a rare, autosomal-recessive neurodegenerative disorder with infant onset, and patients usually die in childhood [1,4,6]. In an INAD cohort with *PLA2G6* mutations previously described by Gregory et al., symptoms began between 5 months and 2.5 years of age [1]. Wu et al. reported that half of INAD patients with a disease course of 2–5 years became vegetative [6]. In this study, the patient’s initial neurological manifestation was cerebellar ataxia at the age of 3 years, which was later accompanied by mental retardation, dystonia, and seizures. The patient survived until the age of 20 years. His clinical phenotype was atypical for INAD; the disease onset and progression were delayed and slower, and there was no indication of truncal hypotonia, neuro-ophthalmologic abnormalities, or fast rhythms on an electroencephalogram throughout the clinical course. Gregory et al. previously described six patients in their patient registry with *PLA2G6* mutation who exhibited variable clinical phenotypes with late onset (average 4.4 years, range 1.5-6.5 years) [1,2]. This phenotype was referred to as atypical neuroaxonal dystrophy (ANAD). The clinical phenotype of our patient might be classified as ANAD. Moreover, patients with a slower disease progression and heterogeneous clinical pictures have been occasionally described in other series of INAD patients [7,8]. Currently, it is speculated that patients with mutant forms of the *PLA2G6* gene display a complete absence of protein, which is associated with a severe INAD profile, whereas patients with compound heterozygous mutations potentially exhibit residual protein function and have a less severe phenotype [18]. Our genetic findings further support this genotype-phenotype correlation.

To the best of our knowledge, there have only been two reports that describe the CNS neuropathology of genetically confirmed patients with a *PLA2G6* gene mutation, as summarized in Table 1[1,8]. The neuropathological features, including neuroaxonal spheroids, cerebellar degeneration, and brain iron accumulation, were described in INAD before the discovery of its causal gene [3]. Moreover, the presence of LB and NFT pathology has been described in neuroaxonal dystrophy with *PLA2G6* gene mutation [1,8]. On the basis of disease onset, patients 1–4 in Table 1 might be classified as ANAD or early onset dystonia-parkinsonism; however, no components of the pathological findings differed between these clinical phenotypes. In the current patient, neuronal loss, LB pathology, NFT pathology, and the presence of axonal spheroids were marked in the limbic system, fronto-temporal lobes, and SN. These pathological findings might be responsible for progressive cortical atrophy, psychomotor regression, and parkinsonism. Iron deposition broadly extended throughout the basal ganglia and midbrain compared to what was predicted from the T2 low-intensity area on MRI. Furthermore, loss of cerebellar neurons, particularly granule cells, was both striking and consistent with the cerebellar ataxia that was diagnosed in the early phase of the disease.

**Table 1:** **Summary of the neuropathological findings in autopsied patients with *PLA2G6 *** **gene mutations in the literature**

**Patient**	**Age at onset**	**Age at death**	**Spheroids in the CNS**	**Spheroids in the PNS**	**Neuronal loss in the cerebellum**	**Accumulation of alpha synuclein**	**Accumulation of tau**	**Brain iron**
**1**[8]	18 y	36 y	+	NA	gc and Pc	+	+	+
**2**[1]	3 y	23 y	+	-	gc and Pc	+	+	+
**3**[8]	childhood	18 y	+	NA	gc and Pc	+	+	+
**4**[8]	14 m	8 y	+	NA	gc and Pc	+	+	+
**5**[8]	infant	8 y	+	NA	NA	+	-	NA
**Our patient**	3 y	20 y	+	-	gc and Pc	+	+	+

Abbreviation: CNS = central nervous system; gc = granule cell; NA = not assessed; Pc = Purkinje cell; PNS = peripheral nervous system.

LB pathology has been identified in all six patients reviewed [1,8]. A recent study reported that LB pathology was not observed in patients with the *PANK2* gene mutation [19]. Moreover, earlier case reports describing neuroaxonal dystrophy or brain iron accumulation with abundant LBs may have been describing patients with *PLA2G6* gene mutations [20-22]. We demonstrated that the morphological, ultrastructural, and biochemical properties of LBs in this patient were identical to those in PD and diffuse Lewy body disease (DLB) patients [12,16,23]. Furthermore, the spatial distribution of LB pathology showed cortical involvement that exceeded that of the end stage of sporadic PD or the diffuse neocortical type of DLB [13,14]. Previous autopsy reports of INAD and ANAD have also described marked cortical involvement of LBs [1,8]. In contrast, the dorsal nucleus of the vagus nerve and olfactory bulbs were mildly affected in our patient, and the cardiac nerve fibers and enteral nerve plexus contained no p-α-synuclein aggregation, although these regions have been described as constant and early affected regions in sporadic PD and DLB [24,25]. The distribution of LB pathology in INAD and ANAD may tend to be more severe in the cerebral cortices compared to the medulla oblongata or peripheral autonomic neurons, which differs from the typical topography observed in sporadic PD and DLB. NFT pathology was another neuropathologial characteristic of interest in patients with INAD and ANAD. In our patient, NFTs and p-tau-positive threads predominantly appeared in the limbic system, which was similar to AD in Braak’s stage IV [15]. However, neither this nor previously reported patients with *PLA2G6* gene mutations exhibited senile plaques, which contrasts with the typical neuropathology observed in AD [15]. Importantly, NFT pathology has been frequently observed in patients with sporadic PD or DLB [26,27], and LBs and NFTs often coexist in the same neurons, particularly those located in the limbic areas [26]. Our double-immunofluorescence results are consistent with findings in sporadic PD and DLB. Thus, further investigation in multiple patients on the association between NFTs and LB pathology and the implications of NFT pathology in INAD and ANAD are required.

The *PLA2G6* gene encodes iPLA2-Via, which is a critical protein in lipid membrane homeostasis [28]. Recent reports using Pla2g6-knockout mice demonstrated the presence of axonal spheroids in which tubulovesicular membranes accumulated [29-32]. In contrast, the pathological mechanism that contributes to LB formation in INAD and ANAD remains to be elucidated. LBs are secondarily present in several situations other than sporadic PD/DLB (e.g., sporadic or familial AD or Niemann-Pick disease type C) or may be incidentally found in healthy elderly individuals [33-36]. However, our neuropathological results and previous studies have demonstrated that LB pathology in patients with *PLA2G6* gene mutations shows a high prevalence and displays an extremely severe phenotype, particularly in the cerebral cortices. These findings suggest that defects in PLA2G6 primarily contribute to the formation of LBs.

## Conclusions

Our results demonstrate the clinical heterogeneity of neuroaxonal dystrophy with *PLA2G6* gene mutations and support a genetic clinical view that compound heterozygous mutations that potentially result in residual protein function are associated with a less severe phenotype. Neuropathologically, CNS involvement with LBs was striking and exhibited a unique topography compared with PD. Thus, further investigations on the process of LB formation caused by loss of *PLA2G6* gene function may provide new insights into the pathological mechanism of neuroaxonal dystrophy and LB formation.

## Consent

Written informed consent was obtained from the patient’s parents for publication of this Case report and any accompanying images. A copy of the written consent is available for review by the Editor-in chief of this journal.

## Competing interests

There are no competing interests in the report.

## Authors’ contributions

YR, MM, and MY performed clinical and pathological analysis. TI, HY, and HH carried out the biochemical and genetic studies and drafted the manuscript. GS, KM, and YG helped to draft the manuscript. All authors read and approved the final manuscript.

## References

[B1] Gregory A Westaway SK Holm IE Kotzbauer PT Hogarth P Sonek S Coryell JC Nguyen TM Nardocci N Zorzi G Rodriguez D Desguerre I Bertini E Simonati A Levinson B Dias C Barbot C Carrilho I Santos M Malik I Gitschier J Hayflick SJ Neurodegeneration associated with genetic defects in phospholipase A_2_ Neurology 2008 1 1402 1409 10.1212/01.wnl.0000327094.67726.28 18799783 PMC2676964

[B2] Gregory A Polster BJ Hayflick SJ Clinical and genetic delineation of neurodegeneration with brain iron accumulation J Med Genet 2009 1 73 80 18981035 10.1136/jmg.2008.061929 PMC2675558

[B3] Seitelberger F Vinken PJ, Bruyn GW, Klawans HL Neuroaxonal dystrophy: its relation to aging and neurological diseases Handbook of Clinical Neurology. Volume 5 1986 Amsterdam: Elsevier 391 415

[B4] Kurian MA Morgan NV MacPherson L Foster K Peake D Gupta R Philip SG Hendriksz C Morton JEV Kingston HM Rosser EM Wassmer E Gissen P Maher ER Phenotypic spectrum of neurodegeneration associated with mutations in the PLA2G6 gene (PLAN) Neurology 2009 1 1623 1629 10.1212/01.wnl.0000310986.48286.8e 18443314

[B5] Morgan NV Westaway SK Morton JE Gregory A Gissen P Sonek S Cangul H Coryell J Canham N Nardocci N Zorzi G Pasha S Rodriguez D Desguerre I Mubaidin A Bertini E Trembath RC Simonati A Schanen C Johnson CA Levinson B Woods CG Wilmot B Kramer P Gitschier J Maher ER Hayflick SJ PLA2G6, encoding a phospholipase A_2_, is mutated in neurodegenerative disorders with high brain iron Nat Genet 2006 1 752 754 10.1038/ng1826 16783378 PMC2117328

[B6] Wu Y Jiang Y Gao Z Wang J Yuan Y Xiong H Chang X Bao X Zhang Y Xiao J Wu X Clinical study and PLA2G6 mutation screening analysis in Chinese patients with infantile neuroaxonal dystrophy Eur J Neurol 2009 1 240 245 10.1111/j.1468-1331.2008.02397.x 19138334

[B7] Nardocci N Zorzi G Farina L Binelli S Scaioli W Ciano C Verga L Angelini L Savoiardo M Bugiani O Infantile neuroaxonal dystrophy: Clinical spectrum and diagnostic criteria Neurology 1999 1 1472 1478 10.1212/WNL.52.7.1472 10227637

[B8] Paisan-Ruitz C Li A Schneider SA Holton JL Johnson R Kidd D Chataway J Bhatia KP Lees AJ Hardy J Revesz T Houlden H Widespread Lewy body and tau accumulation in childhood and adult onset dystonia-parkinsonism cases with PLA2G6 mutations Neurobiol Aging 2012 1 814 823 10.1016/j.neurobiolaging.2010.05.009 20619503 PMC3657696

[B9] Yoshino H Tomiyama H Tachibana N Yoshino H Tomiyama H Tachibana N Ogaki K Li Y Funayama M Hashimoto T Takashima S Hattori N Phenotypic spectrum of patients with PLA2G6 mutation and PARK14-linked parkinsonism Neurology 2010 1 1356 1361 10.1212/WNL.0b013e3181f73649 20938027

[B10] Ikeuchi T Kakita A Shiga A Kasuga K Kaneko H Tan CF Idezuka J Wakabayashi K Onodera O Iwatsubo T Nishizawa M Takahashi H Ishikawa A Homozygous and heterozygous patients for *SNCA* duplication in family with parkinsonism and dementia Arch Neurol 2008 1 514 519 10.1001/archneur.65.4.514 18413475

[B11] Kaneko H Kakita A Kasuga K Nozaki H Ishikawa A Miyashita A Kuwano R Ito G Iwatsubo T Takahashi H Nishizawa M Onodera O Sisodia SS Ikeuchi T Enhanced accumulation of phosphorylated α-synuclein and elevated Aβ42/40 ratio caused by expression of the presenilin-1 delta T440 mutant associated with familial Lewy body disease and variant Alzheimer disease J Neurosci 2007 1 13092 13097 18045903 10.1523/JNEUROSCI.4244-07.2007 PMC6673391

[B12] Fujiwara H Hasegawa M Dohmae N Kawashima A Masliah E Goldberg MS Shen J Takio K Iwatsubo T α-synuclein is phosphorylated in synucleinopathy lesions Nat Cell Biol 2002 1 160 164 11813001 10.1038/ncb748

[B13] Braak H Del Tredici K Rüb U de Vos RA Jansen Steur EN Braak E Staging of brain pathology related to sporadic Parkinson’s disease Neurobiol Aging 2003 1 197 211 10.1016/S0197-4580(02)00065-9 12498954

[B14] McKeith IG Dickson DW Lowe J Emre M O’Brien JT Feldman H Cummings J Duda JE Lippa C Perry EK Aarsland D Arai H Ballard CG Boeve B Burn DJ Costa D Del Ser T Dubois B Galasko D Gauthier S Goetz CG Gomez-Tortosa E Halliday G Hansen LA Hardy J Iwatsubo T Kalaria RN Kaufer D Kenny RA Korczyn A Kosaka K Lee VM Lees A Litvan I Londos E Lopez OL Minoshima S Mizuno Y Molina JA Mukaetova-Ladinska EB Pasquier F Perry RH Schulz JB Trojanowski JQ Yamada M Diagnosis and management of dementia with Lewy bodies: third report of the DLB Consortium Neurology 2005 1 1863 1872 10.1212/01.wnl.0000187889.17253.b1 16237129

[B15] Braak H Alafuzoff I Arzberger T Kretzschmar H Tredici KD Staging of Alzheimer’s disease-associated neurofibrillary pathology using paraffin sections and immunocytochemistry Acta Neuropathol 2006 1 389 404 10.1007/s00401-006-0127-z 16906426 PMC3906709

[B16] Kosaka K Lewy bodies in cerebral cortex, report of three cases Acta Neuropathol (Berl) 1978 1 127 134 10.1007/BF00690978 654884

[B17] Ma Z Wang X Nowatzke W Ramanadham S Turk J Human pancreatic islets express mRNA species encoding two distinct catalytically active isoforms of group VI phopholipase A2 (iPLA2) that arise from an exon-skipping mechanism of alternative splicing of the transcript from the iPLA2 gene on chromosome 22q13.1 J Biol Chem 1999 1 9607 9616 10.1074/jbc.274.14.9607 10092647 PMC3715997

[B18] Tonelli A Romaniello R Grasso R Cavallini A Righini A Bresolin N Borgatti R Bassi MT Novel splice-site mutations and a large intragenic deletion in PLA2G6 associated with a severe and rapidly progressive form of infantile neuroaxonal dystrophy Clin Genet 2010 1 432 440 10.1111/j.1399-0004.2010.01417.x 20584031

[B19] Li A Paudel R Johnson R Courtney R Lees AJ Holton JL Hardy J Revesz T Houlden H Pantothenate kinase-associated neurodegeneration is not a synucleinopathy Neuropathol Appl Neurobiol 2013 1 121 131 10.1111/j.1365-2990.2012.01269.x PMC3712463 22416811

[B20] Wakabayashi K Fukushima T Koide R Horikawa Y Hasegawa M Watanabe Y Noda T Eguchi I Morita T Yoshimoto M Iwatsubo T Takahashi H Juvenile-onset generalized neuroaxonal dystrophy (Hallervorden-Spatz disease) with diffuse neurofibrillary and Lewy body pathology Acta Neuropathol (Berl) 2000 1 331 336 10.1007/s004010050049 10663979

[B21] Wakabayashi K Yoshimoto M Fukushima T Koide R Horikawa Y Morita T Takahashi H Widespread occurrence of α-synuclein/NACP immunoreactive neuronal inclusions in juvenile and adult-onset Hallervorden–Spatz disease with Lewy bodies Neuropathol Appl Neurobiol 1999 1 363 368 10.1046/j.1365-2990.1999.00193.x 10564525

[B22] Neumann M Adler S Schlüter O Kremmer E Benecke R Kretzschmar HA α-Synuclein accumulation in a case of neurodegeneration with brain iron accumulation type 1 (NBIA-1, formerly Hallervorden-Spatz syndrome) with wide spread cortical and brainstem-type Lewy bodies Acta Neuropathol (Berl) 2000 1 568 574 10.1007/s004010000224 11045680

[B23] Forno LS Neuropathology of Parkinson’s disease J Neuropathol Exp Neurol 1996 1 259 272 10.1097/00005072-199603000-00001 8786384

[B24] Hawkes CH Tredici KD Braak H Review: Parkinson’s disease: a dual-hit hypothesis Neuropathol Appl Neurobiol 2007 1 599 614 10.1111/j.1365-2990.2007.00874.x 17961138 PMC7194308

[B25] Orimo S Takahashi A Uchihara T Mori F Kakita A Wakabayashi K Takahashi H Degeneration of cardiac sympathetic nerve begins in the early disease process of Parkinson’s disease Brain Pathol 2007 1 24 30 10.1111/j.1750-3639.2006.00032.x 17493034 PMC8095543

[B26] Iseki E Marui W Kosaka K Frequent coexistence of Lewy bodies and neurofibrillary tangles in the same neurons of patients with diffuse Lewy body disease Neurosci Lett 1999 1 9 12 10.1016/S0304-3940(99)00178-0 10327193

[B27] Ishizawa T Mattila P Davies P Wang D Dickson DW Colocalization of tau and alpha-synuclein epitopes in Lewy bodies J Neuropathol Exp Neurol 2003 1 389 397 12722831 10.1093/jnen/62.4.389

[B28] Baburina I Jakowski S Cellular responses to excess phospholipid J Biol Chem 1999 1 9400 9408 10092620 10.1074/jbc.274.14.9400

[B29] Beck G Sugiura Y Shinzawa K Kato S Setou M Tsujimoto Y Sakoda S Sumi-Akamaru H Neuroaxonal dystrophy in calcium-independent phospholipase A_2_β deficiency results from insufficient remodeling and degeneration of mitochondrial and presynaptic membranes J Neurosci 2011 1 11411 11420 10.1523/JNEUROSCI.0345-11.2011 21813701 PMC6623367

[B30] Malik I Turk J Mancuso DJ Montier L Wohltmann M Wozniak DF Schmidt RE Gross RW Kotzbauer PT Disrupted membrane homeostasis and accumulation of ubiquitinated proteins in a mouse model of infantile neuroaxonal dystrophy caused by PLA2G6 mutations Am J Pathol 2008 1 406 416 10.2353/ajpath.2008.070823 18202189 PMC2312364

[B31] Shinzawa K Sumi H Ikawa M Matsuoka Y Okabe M Sakoda S Tsujimoto Y Neuroaxonal dystrophy caused by group VIA phospholipase A_2_ deficiency in mice: a model of human neurodegenerative disease J Neurosci 2008 1 2212 2220 10.1523/JNEUROSCI.4354-07.2008 18305254 PMC6671850

[B32] Wada H Yasuda T Miura I Watabe K Sawa C Kamijuku H Kojo S Taniguchi M Nishino I Wakana S Yoshida H Seino K Establishment of an improved mouse model for infantile neuroaxonal dystrophy that shows early disease onset and bears a point mutation in Pla2g6 Am J Pathol 2009 1 2257 2263 10.2353/ajpath.2009.090343 19893029 PMC2789634

[B33] Hamilton RL Lewy bodies in Alzheimer’s disease: A neuropathological review of 145 cases using α-synuclein immunohistochemistry Brain Pathol 2000 1 378 384 10885656 10.1111/j.1750-3639.2000.tb00269.x PMC8098522

[B34] Rosenberg CK Pericak-Vance MA Saunders AM Gilbert JR Gaskell PC Hulette CM Lewy body and Alzheimer pathology in a family with the amyloid-β precursor protein APP717 gene mutation Acta Neuropathol (Berl) 2000 1 145 152 10.1007/s004019900155 10963361

[B35] Saito Y Ruberu NN Sawabe M Arai T Kazama H Hosoi T Yamanouchi H Murayama S Lewy body-related α-synucleinopathy in aging J Neuropathol Exp Neurol 2004 1 742 749 15290899 10.1093/jnen/63.7.742

[B36] Saito Y Suzuki K Hulette CM Murayama S Aberrant phosphorylation of α-synuclein in human Niemann-Pick type C1 disease J Neuropathol Exp Neurol 2004 1 323 328 15099022 10.1093/jnen/63.4.323

